# Novel insights into the mechanism of well-ordered assembly of bacterial flagellar proteins in *Salmonella*

**DOI:** 10.1038/s41598-018-20209-3

**Published:** 2018-01-29

**Authors:** Yumi Inoue, Yusuke V. Morimoto, Keiichi Namba, Tohru Minamino

**Affiliations:** 1 Graduate School of Frontier Biosciences, Osaka University, 1-3 Yamadaoka, Suita, Osaka 565-0871 Japan; 2 Quantitative Biology Center, RIKEN, 6-2-3 Furuedai, Suita, Osaka 565-0874 Japan; 3 Department of Bioscience and Bioinformatics, Faculty of Computer Science and Systems Engineering, Kyushu Institute of Technology, 680-4 Kawazu, Iizuka, Fukuoka 820-8502 Japan

## Abstract

The FliI ATPase of the flagellar type III protein export apparatus forms the FliH_2_FliI complex along with its regulator FliH. The FliH_2_FliI complex is postulated to bring export substrates from the cytoplasm to the docking platform made of FlhA and FlhB although not essential for flagellar protein export. Here, to clarify the role of the FliH_2_FliI complex in flagellar assembly, we analysed the effect of FliH and FliI deletion on flagellar protein export and assembly. The hook length was not controlled properly in the ∆*fliH-fliI flhB*(P28T) mutant compared to wild-type cells, whose hook length is controlled to about 55 nm within 10% error. The FlhA(F459A) mutation increased the export level of the hook protein FlgE and the ruler protein FliK by about 10-fold and 3-fold, respectively, and improved the hook length control in the absence of FliH and FliI. However, the ∆*fliH-fliI flhB*(P28T) *flhA*(F459A) mutant did not produce flagellar filaments efficiently, and a large amount of flagellin monomers were leaked out into the culture media. Neither the hook length control nor flagellin leakage was affected by the FlhB(P28T) and FlhA(F459A) mutations. We will discuss a hierarchical protein export mechanism of the bacterial flagellum.

## Introduction

The bacterial flagellum, which is responsible for motility, is composed of basal body rings and an axial structure consisting of the rod (FliE, FlgB, FlgC, FlgF, FlgG), the hook (FlgE), the hook-filament junction (FlgK, FlgL), the filament (FliC) and the filament cap (FliD). The assembly of the axial structure begins with the rod, followed by the hook, the hook-filament junction, the filament cap in this order, and finally the filament with the help of the filament cap. Axial component proteins are transported via a type III protein export apparatus located at the flagellar base from the cytoplasm to the distal end of the growing structure for their assembly^1–3^.

**Figure 1:**
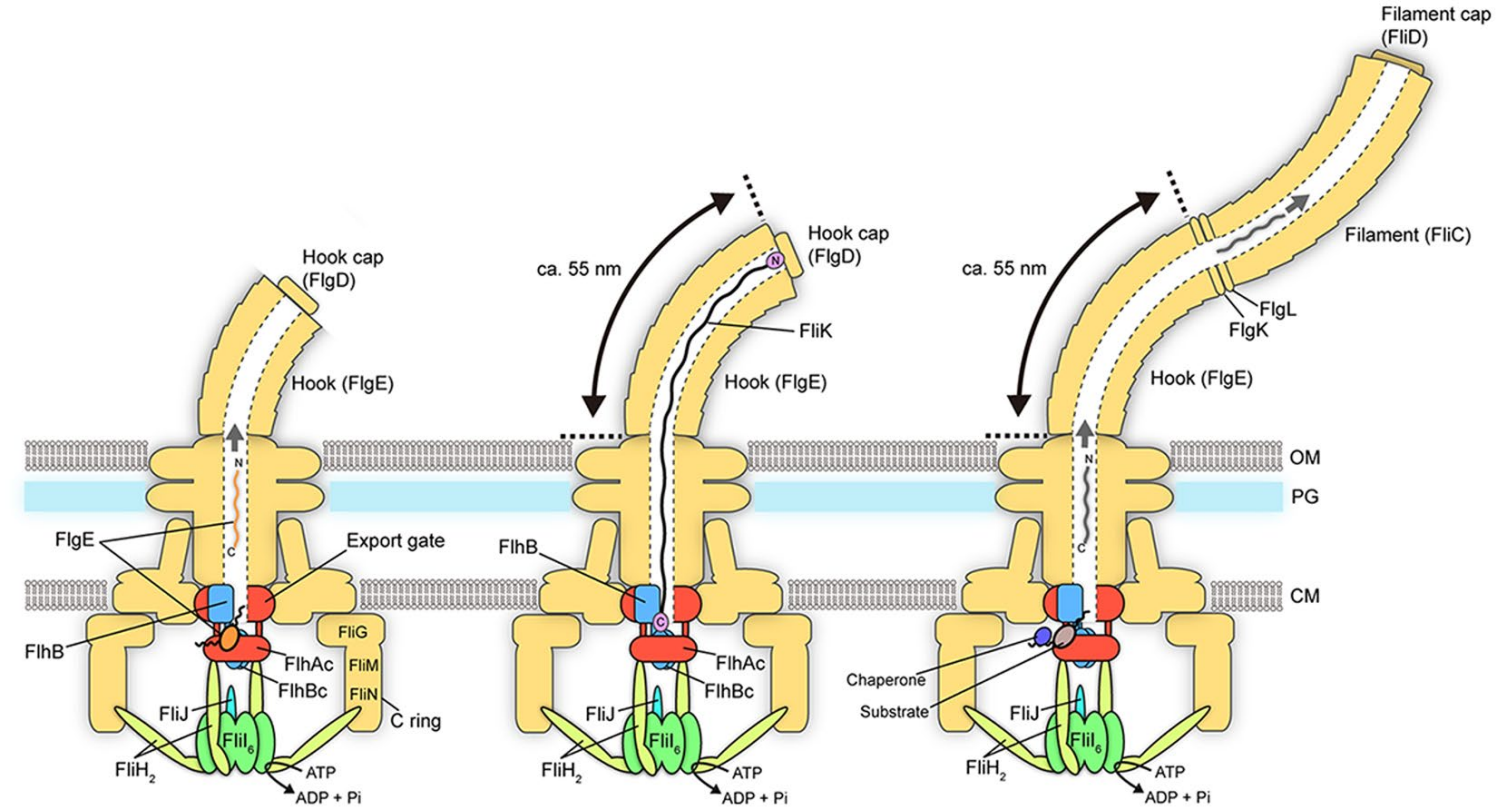
Export switching mechanism of the flagellar type III protein export apparatus. The flagellar type III protein export apparatus, which is composed of a transmembrane export gate complex (indicated as Export gate) and a cytoplasmic ATPase ring complex consisting of FliH, FliI and FliJ, transport the hook protein FlgE during hook assembly (left panel). FliK is also exported during hook assembly and acts as a ruler to measure the hook length. When the hook length reaches to about 55 nm, the C-terminal domain of FliK binds to the C-terminal domain of a transmembrane export gate protein FlhB (FlhB_C_), switching export specificity of the export apparatus (middle panel). As a result, the export apparatus terminates the export of FlgE and FliK and initiates the export of filament-type export substrates such as FlgK, FlgL, FliC and FliD (right panel). The C-terminal cytoplasmic domain of FlhA (FlhA_C_) provides binding sites for the filament-type substrates in complex with their cognate export chaperones.

The flagellar type III protein export apparatus is composed of a transmembrane export gate complex made of FlhA, FlhB, FliP, FliQ and FliR and a cytoplasmic ATPase complex consisting of FliH, FliI and FliJ^4–6^. These proteins are highly homologous to components of the injectisome of pathogenic bacteria, which directly injects virulence effector proteins into their host cells^7^. The flagellar protein export apparatus switches its substrate specificity from proteins needed for the structure and assembly of the hook (FlgD, FlgE, FliK) (hook-type substrates) to those responsible for filament formation (FlgK, FlgL, FliD, FliC) (filament-type substrates) upon completion of hook assembly, thereby determining the hook length at about 55 nm in *Salmonella enterica* (thereafter referred to *Salmonella*) (Fig. 1)^8,9^. Four flagellar proteins, namely FliK, FlhA, FlhB and Flk, are responsible for this switching mechanism. FliK is recognized as a hook-type substrate by the flagellar protein export apparatus to be transported into the central channel inside the growing hook structure^10^ and acts as an infrequent ruler to measure the hook length (Fig. 1, middle panel)^11^. The C-terminal cytoplasmic domains of FlhA (FlhA_C_) and FlhB (FlhB_C_), which are transmembrane proteins of the export apparatus, form a docking platform for the cytoplasmic ATPase complex, type III export chaperones and export substrates, and coordinate flagellar protein export with assembly^5,12–17^. Genetic analyses have shown that an interaction between the C-terminal domain of FliK (FliK_C_) and FlhB_C_ is responsible for the substrate specificity switch^9,18^. This is supported by recent photo-crosslinking experiments^19^. Flk interferes with the interaction of FliK_C_ with FlhB_C_ until the hook reaches its mature length^20,21^.

The cytoplasmic ATPase ring complex of the export apparatus is formed by six copies of the FliH homo-dimer, six copies of the FliI ATPase and one copy of FliJ^22–24^ and is located at the flagellar base through interactions of the extreme N-terminal region of FliH with FlhA and a C ring protein FliN (Fig. 1)^25–28^. This ATPase ring is not essential for flagellar protein export^29,30^ but ensures robust and efficient energy coupling of protein export during flagellar assembly^31^. The FliH dimer and the FliI ATPase also exist as the FliH_2_FliI complex^32^ and binds to export substrates in complex with their cognate type III export chaperones^33,34^. Since FliI-YFP exchanges between the flagellar basal body and the cytoplasmic pool, the FliH_2_FliI complex is postulated to bring export substrates and type III export chaperone–substrate complexes from the cytoplasm to the FlhA_C_-FlhB_C_ platform^28^.

Many suppressor mutants have been isolated from a *Salmonella* ∆*fliH-fliI* mutant. The FlhB(P28T) mutation is one of the suppressor mutations and recovers the export efficiency of export substrates to a considerable degree, thereby allowing more than 80% of the ∆*fliH-fliI* mutant cells to form a few flagella in the absence of FliH and FliI^29,35^. The level of FlgE secreted by the ∆*fliH-fliI flhB*(P28T) mutant (thereafter referred to ∆fliH-fliI flhB*) is about 10-fold less than the wild-type level whereas that of the hook-capping protein FlgD is almost at the wild-type level, raising the possibility that FlgE may require FliH and FliI for its more efficient export and assembly compared to the export of FlgD^29^. To clarify this, we isolated hook-basal bodies (HBBs) from the ∆fliH-fliI flhB* mutant cells and measured the hook length by electron microscopy. We also quantitatively analysed the efficiency of filament assembly. We show that the ∆fliH-fliI flhB* mutant cells cannot properly control the hook length. We also show that the FlhA(F459A) mutation improves the hook assembly and its length control by the ∆fliH-fliI flhB* mutant cells but not filament assembly at all.

## Results

### Effect of depletion of FliH and FliI on hook length control

**Figure 2:**
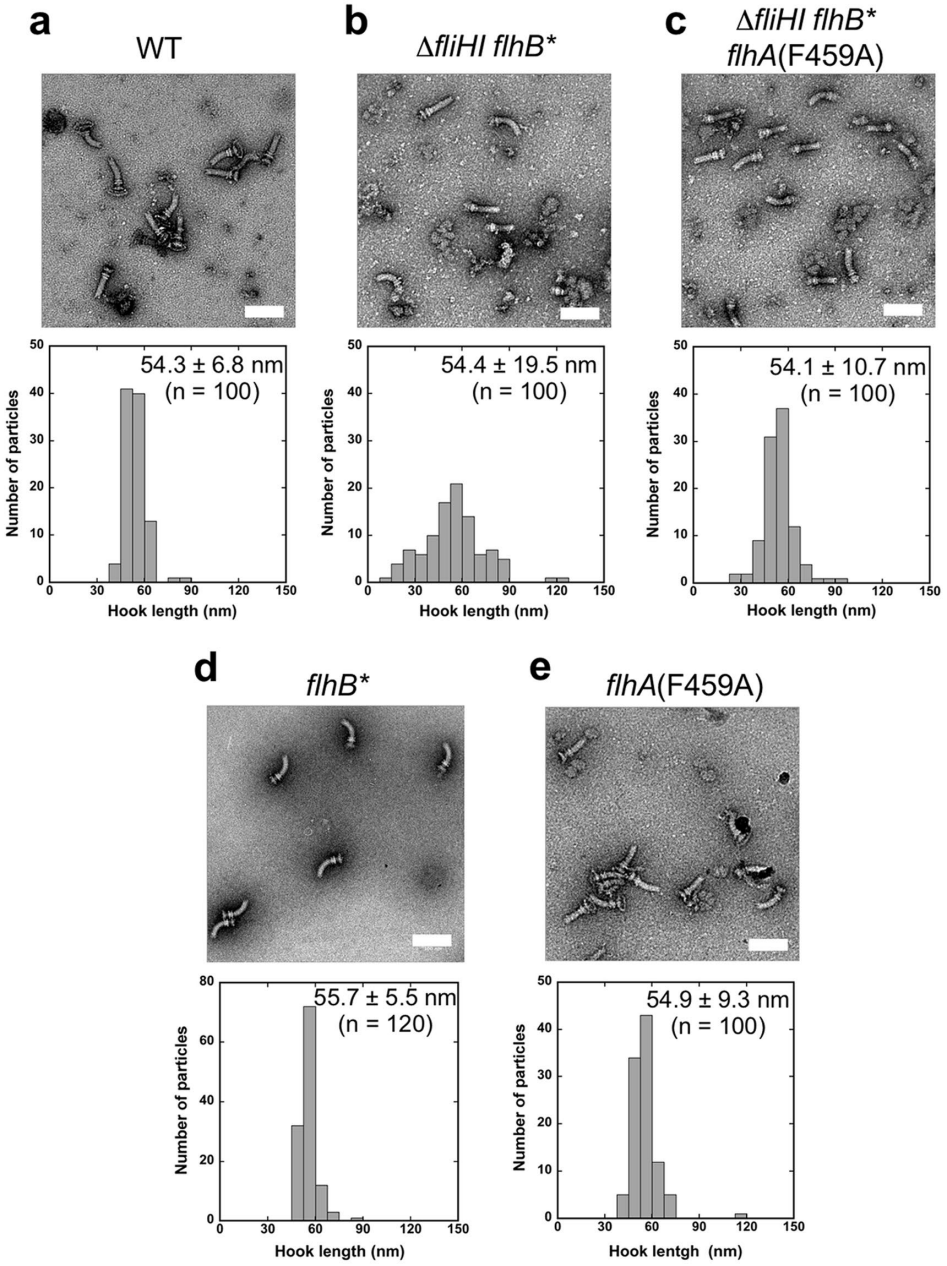
Effect of FliH and FliI deletion on the hook length control of the *Salmonella* flagellum. Electron micrographs of HBBs and histograms of hook length distribution of (**a**) SJW1103 (WT), (**b**) MMHI0117 (∆fliHI flhB*), (**c**) MMHI0117-1 [∆fliHI flhB* flhA(F459A)], (**d**) MMB017 (flhB*) and (**e**) MMA459 [*flhA*(F459A)]. Hook length was measured on electron micrographs recorded at a magnification of x5, 500, which corresponds to 2.75 nm per pixel. Scale bar, 100 nm.

It has been suggested that FliH and FliI may be required for efficient export of FlgE during hook-basal body (HBB) assembly in *Salmonella*^29^. We used a ∆fliH-fliI flhB* mutant^29^ to study the effect of FliH and FliI deletion on the hook length. A *Salmonella* wild-type strain has several flagella whereas the ∆fliH-fliI flhB* mutant produces a few flagella^29,35^. Therefore, we isolated intact flagella from the wild-type and ∆fliH-fliI flhB* mutant cells by 20–50% sucrose density-gradient ultracentrifugation and performed acidic treatment to depolymerize the filaments from the HBBs to measure the hook length. After ultracentrifugation, the HBBs were negatively stained with 2% uranyl acetate and were observed by electron microscopy (Fig. 2). The hook length of the ∆fliH-fliI flhB* mutant was 54.4 ± 19.5 (mean ± SD, n = 100) nm, where the average length was nearly the same as that of the wild-type strain (54.3 ± 6.8 nm, n = 100) (Fig. 2a and b). However, while the majority of wild-type hook length was distributed within a range from 50 to 60 nm, the hook length distribution of the ∆fliH-fliI flhB* mutant was much broader. The hooks isolated from the ∆fliH-fliI flhB* mutant cells not only showed a significantly larger population in a much longer range from 60 to 90 nm but also showed a small but significant population in a much shorter range from 15 to 35 nm. This suggests that the ∆fliH-fliI flhB* mutant cannot properly control the hook length even in the presence of the FliK ruler.

### Effect of FlhA mutations on flagellar protein export

**Figure 3:**
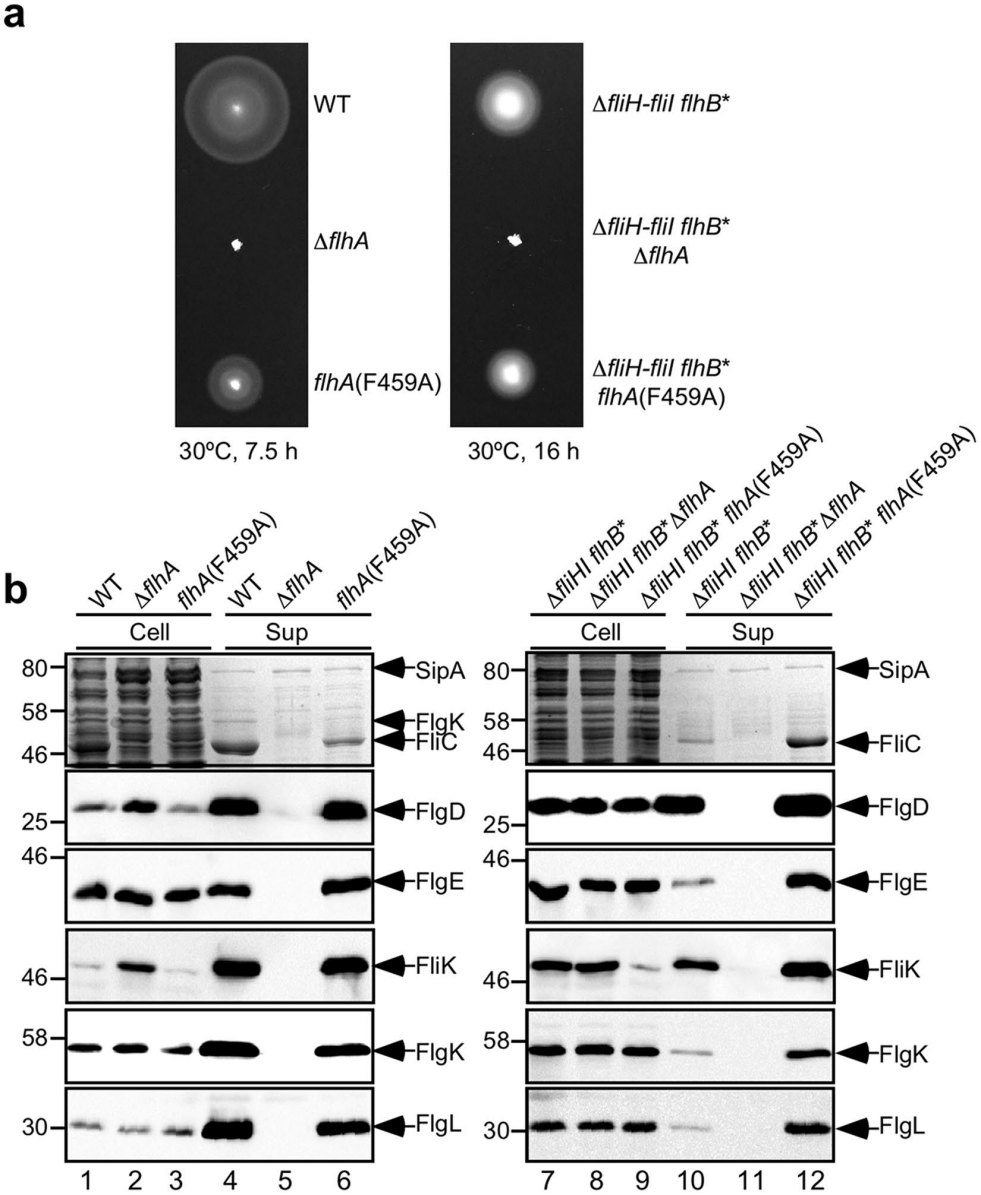
Effect of the FlhA(F459A) mutation on flagellar protein export. (**a**) Effect of the FlhA(F459A) mutation on motility. Motility of SJW1103 (WT), NH001 (∆*flhA*), MMA459 [*flhA*(F459A)], MMHI0117 (∆fliHI flhB*), NH004 (∆fliHI flhB* ∆*flhA*) and MMHI0117-1 [∆fliHI flhB* flhA(F459A)] in soft agar. Plates were incubated at 30 °C. (**b**) Effect of the FlhA(F459A) mutation on flagellar protein export in the presence (left panels) and absence (right panels) of FliH and FliI. Whole cell proteins (Cell) and culture supernatant fractions (Sup) were prepared from the above strains. An 8 μl solution of each protein sample, which was normalized to an optical density of OD_600_, was subjected to SDS-PAGE and was analyzed by CBB staining (first row) and immunoblotting with polyclonal anti-FlgD (second row), anti-FlgE (third row), anti-FliK (fourth row), anti-FlgK (fifth row) or anti-FlgL (sixth row) antibody. CBB-stained gels and immunoblots were cropped from original image data shown in Fig. S5 in the Supplemental information and were processed using Photoshop CS6. The positions of molecular mass markers (kDa) are indicated on the left. SipA, which is secreted by the SPI-1 virulence-associated type III secretion apparatus^51^, serves as a loading control.

The D456V, F459A and T490M mutations in FlhA_C_ have been identified as bypass mutations of the FlgN defect in the ∆fliH-fliI flhB* ∆*flgN* mutant^15^. FlgN acts as a flagellar type III export chaperone specific for FlgK and FlgL to facilitate the docking of FlgK and FlgL to FlhA_C_ for their efficient protein transport^15,36^. However, these mutations considerably reduce the binding affinity of FlhA_C_ for FlgN^15^, raising the question of how these FlhA mutations enhance the export of FlgK and FlgL by the ∆fliH-fliI flhB* ∆*flgN* mutant. To clarify this question, we replaced the wild-type *flhA* gene of the ∆fliH-fliI flhB* mutant by the *flhA*(F459A) allele using the λ Red homologous recombination system^37^ and analyzed motility of and flagellar protein export by the ∆fliH-fliI flhB* *flhA*(F459A) mutant (Fig. 3). In agreement with a previous report^16^, the FlhA(F459A) mutation reduced motility in the presence of FliH and FliI (Fig. 3a, left panel). Quantitative measurements of substrate secretion levels by measuring each band intensity revealed that the FlhA(F459A) mutation reduced the secretion levels of FlgK, FlgL and FliC by about 2-, 2- and 4-fold, respectively, in the presence of FliH and FliI (Fig. 3b, left panel, 1st, 5th and 6th rows, compared lanes 4 and 6), but did not affect those of FlgD, FlgE and FliK (2nd, 3rd and 4th rows, compared lanes 4 and 6). In the ∆fliH-fliI flhB* mutant, the F459A mutation increased the secretion levels of FlgD, FlgE and FliK by about 1.5-, 10- and 3-fold, respectively (Fig. 3b. right panel, 2nd, 3rd and 4th rows, compared between lanes 10 and 12). Whole cellular levels of FlgD and FlgE in the ∆fliH-fliI flhB* *flhA*(F459A) mutant were almost the same as those in the ∆*fliH-fliI flhB*(P28T) mutant (2nd and 3rd rows, lanes 7 and 9) whereas the cellular level of FliK was much lower in the ∆fliH-fliI flhB* *flhA*(F459A) mutant than that seen in the ∆fliH-fliI flhB* mutant (4th row, lanes 7 and 9). Consistently, this FlhA(F459A) mutation increased the probability of hook formation from about 41 to 96%. The hook length of the ∆fliH-fliI flhB* *flhA*(F459A) mutant was 54.1 ± 10.7 nm (n = 100), which was almost the same as the wild-type hook length (Fig. 2c). The FlhB(P28T) and FlhA(F459A) mutations did not affect the hook length control by themselves (Fig. 2d and e). Since the FlhA(F459A) mutation did not affect the whole cellular level of FlgE, we suggest that the FlhA(F459A) mutation increases the export of FlgE and FliK by about 10-fold and 3-fold, respectively, thereby improving the hook assembly and its length control in the absence of FliH and FliI.

In contrast to the *flhA*(F459A) mutant cells, the levels of FlgK, FlgL and FliC secreted by the ∆fliH-fliI flhB* *flhA*(F459A) mutant were about 10-fold higher than those by the ∆fliH-fliI flhB* mutant (Fig. 3b. right panel, 1st, 5th and 6th rows, lanes 10 and 12). It has been shown that filament-type protein export occurs only after completion of hook assembly^38^. Since we found that FlhA(F459A) mutation increased the probability of hook formation from about 41 to 96%, we suggest that the increased probability of hook formation by the FlhA(F459A) mutation increases the substrate specificity switching frequency of the flagellar type III protein export apparatus, thereby increasing the secretion levels of FlgK, FlgL and FliC. We obtained essentially the same results with the ∆fliH-fliI flhB* *flhA*(D456V) and ∆fliH-fliI flhB* *flhA*(T490M) mutants (Fig. S1). These results suggest that the D456V, F459A and T490M mutations in FlhA_C_ significantly enhance the protein export activity of the export gate complex in the absence of FliH and FliI.

### Effect of FlgE and FliK over-expression on the hook length control in the presence and absence of FliH and FliI

**Figure 4:**
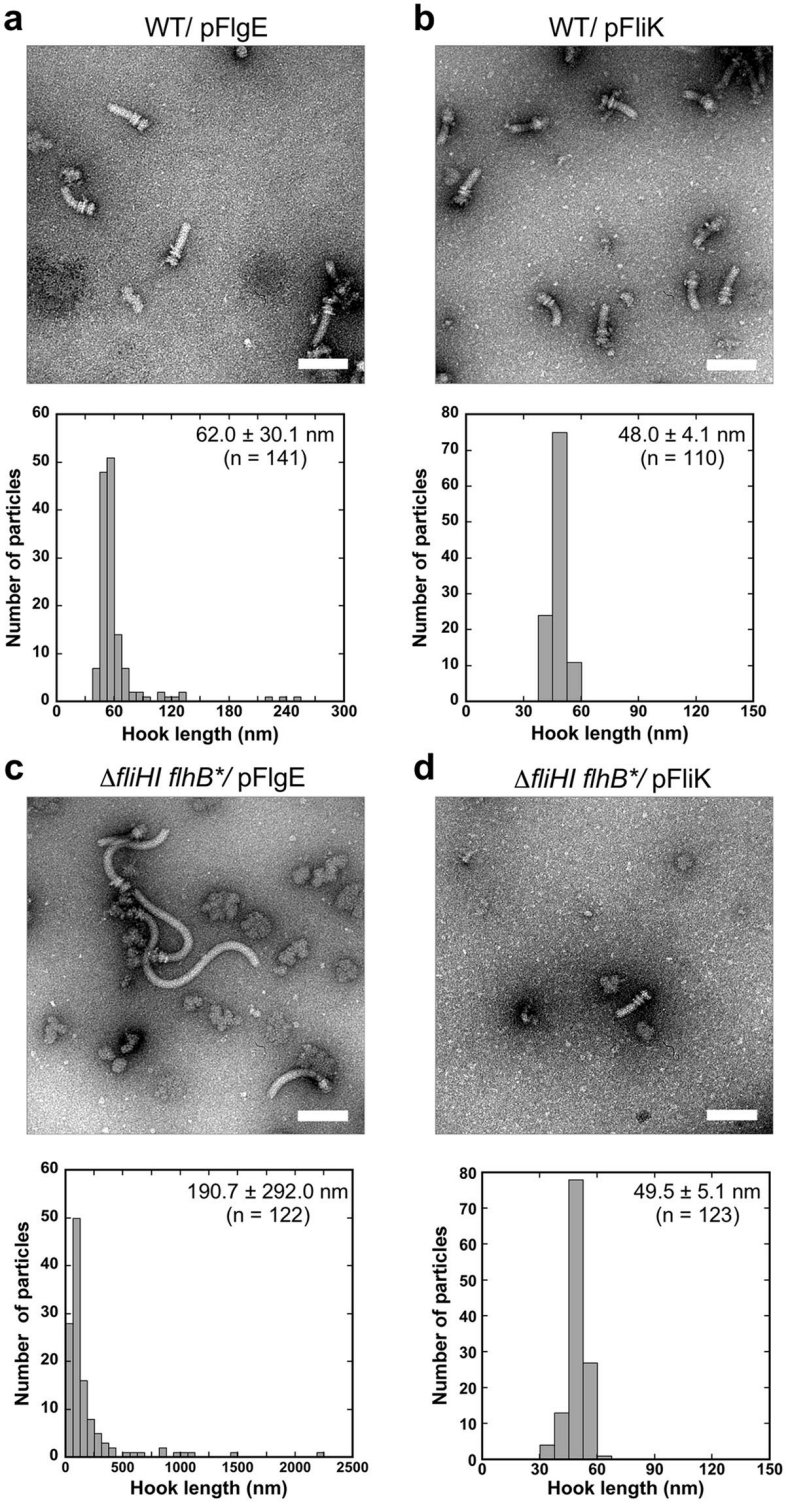
Effect of FlgE and FliK over-expression on the hook length control in the presence and absence of FliH and FliI. Electron micrographs of HBBs and histograms of hook length distribution of (**a**) SJW1103 transformed with pNM001 (WT/pFlgE), (**b**) SJW1103 carrying pMMIK100 (WT/pFliK), (**c**) MMHI0117 harbouring pNM001 (∆fliHI flhB*/pFlgE) and (**d**) MMHI0117 harbouring pMMIK100 (∆fliHI flhB*/pFliK). Scale bar, 100 nm.

Over-expression of FliK in wild-type cells slightly shortens the hooks compared to the wild-type length^39^. In contrast, when the expression level of FliK is reduced, the cell produces polyhooks, sometimes with filaments attached, probably because the probability of FliK measuring the growing hook length is decreased^39^. Longer hooks are frequently observed when FlgE is overproduced in the wild-type^40^. These observations suggest that the balance between the export levels of FlgE and FliK affects the hook length. To test how much the hook length control is affected by the over-expression of FlgE and FliK in the ∆fliH-fliI flhB* mutant, we measured the hook length of the ∆fliH-fliI flhB* mutant cells transformed with a pTrc99AFF4-based plasmid encoding FlgE or FliK (Fig. 4).

When FlgE or FliK was over-expressed in wild-type cells, the whole cellular and secretion levels of FlgE and FliK were increased considerably (Fig. S2a, 2nd and 3rd row). The hook length of wild-type cells overproducing FlgE or FliK was 62.0 ± 30.1 (n = 141) and 48.0 ± 4.1 nm (n = 110), respectively (Fig. 4a and b), in agreement with previous reports^39,40^. When FlgE was over-expressed in the ∆fliH-fliI flhB* mutant (Fig. S2b, 2nd row), much longer polyhooks were frequently observed, and the hook length was 190.7 ± 292.0 nm (n = 122) (Fig. 4c). Interestingly, while the levels of FlgD and FliK secreted by the ∆fliH-fliI flhB* mutant over-expressing FlgE were reduced by only about 1.5-fold compared to the vector control (Fig. S2b. 1st and 3rd rows, lanes 4 and 5), over-expression of FliK reduced the export of FlgE by about 5-fold (Fig. S2. 2nd row, lanes 4 and 6) and so inhibited hook polymerization considerably. As a result, the hook length of the ∆*fliH-fliI flhB*(P28T) mutant cells over-expressing FliK was 49.5 ± 5.1 nm (n = 123) (Fig. 4d).

Over-expression of FlgE or FliK affected the secretion levels of other hook type substrates in both wild-type and ∆fliH-fliI flhB* mutant cells (Fig. S2), raising the possibility that the over-expression of these two proteins might affect the substrate specificity switching efficiency. To clarify this, we analysed the secretion levels of filament-type export substrates such as FlgK (Fig. S2. 4th row), FlgL (5th row) and FliC (6th row) by immunoblotting. Over-expression of FlgD or FliK in wild-type cells did not affect the secretion levels of FlgK, FlgL and FliC (Fig. S2a), indicating that the over-expression of these two proteins does not reduce the switching probability of the flagellar type III protein export apparatus in the wild-type. When FliK was over-expressed in the ∆fliH-fliI flhB* mutant, the secretion levels of FlgK, FlgL and FliC were reduced considerably (Fig. S2b, lane 6), indicating that the switching efficiency of the protein export apparatus was reduced significantly. However, the over-expression of FlgE did not affect the secretion levels of FlgK, FlgL and FliC in the ∆fliH-fliI flhB* mutant (Fig. S2b, lane 5). These results suggest that an excess amount of FliK directly interferes with the export of FlgE in the ∆fliH-fliI flhB* mutant, thereby inhibiting hook polymerization considerably.

### Effect of FliH and FliI deletion on flagellar filament formation

**Figure 5:**
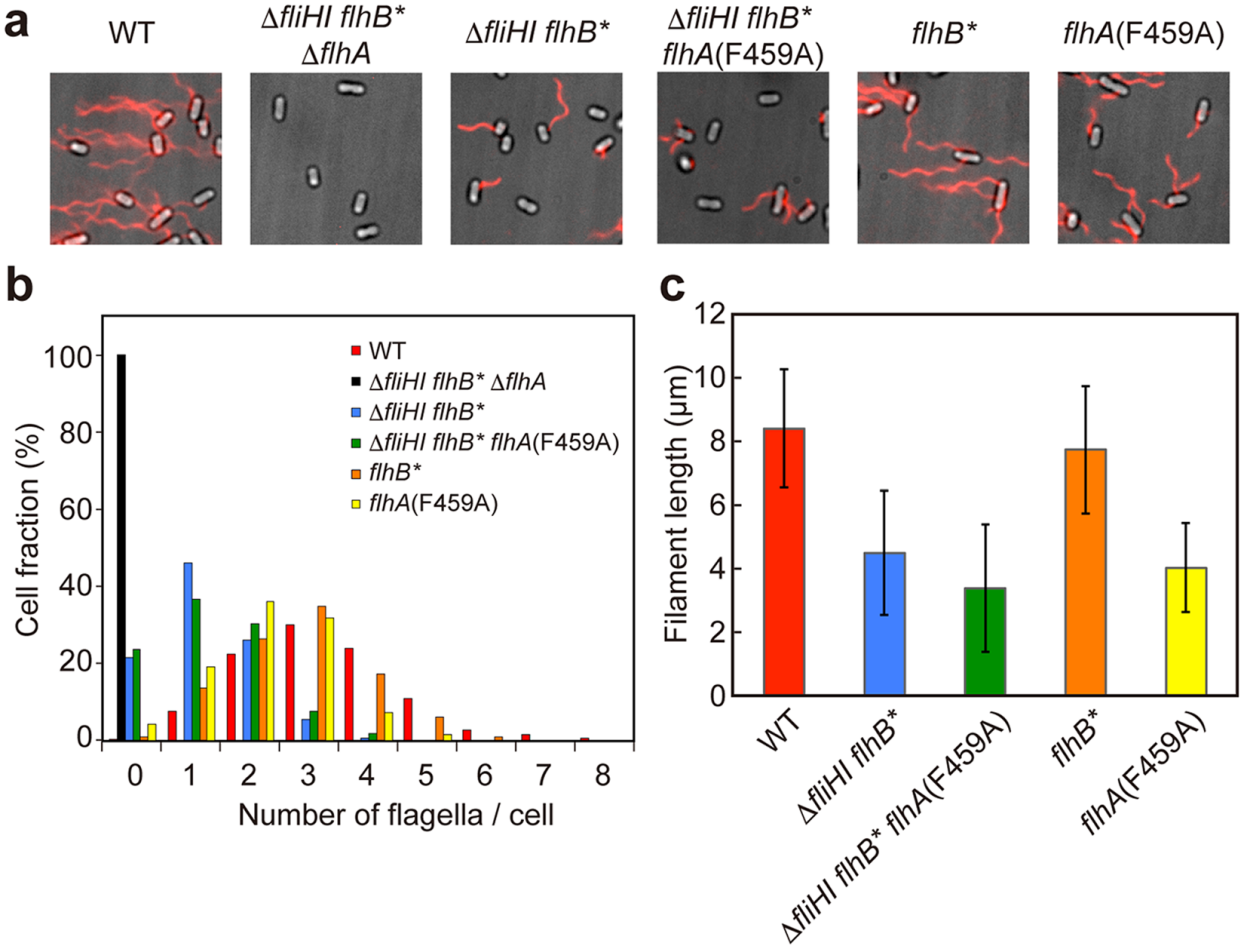
Effect of FliH and FliI deletion on the number and length of the flagellar filaments. (**a**) Fluorescent images of SJW1103 (WT), NH004 (∆fliHI flhB* ∆*flhA*), MMHI0117 (∆fliHI flhB*), MMHI0117-1 [∆fliHI flhB* flhA(F459A)], MMB017 (flhB*) and MMA459 [*flhA*(F459A)]. The flagellar filaments were labeled with Alexa Fluor 594. The fluorescence images of the filaments labeled with Alexa Fluor 594 (red) were merged with the bright field images of the cell bodies. (**b**) Distribution of the number of the flagellar filaments. The filaments were counted for more than 200 cells of each strain. (**c**) Measurements of the length of the flagellar filaments. Filament length is the average of more than 100 cells for each strain, and vertical lines are standard deviations.

We found that the motility of the ∆fliH-fliI flhB* *flhA*(F459A) mutant was worse than that of the ∆fliH-fliI flhB* mutant (Fig. 3a, right panel), although the secretion levels of filament-type substrates were about 10-fold higher than those secreted by the ∆fliH-fliI flhB* mutant (Fig. 3b). To test if the ∆fliH-fliI flhB* *flhA*(F459A) mutant does not form flagellar filaments efficiently, we labeled the filaments with a fluorescent dye (Fig. 5a) and quantitatively analyzed the number and length of the filaments (Fig. 5b and c). The number of the filaments produced by wild-type cells ranged from 1 to 8 with an average of 3.3 ± 1.4 (Fig. 5b). About 80% of the ∆fliH-fliI flhB* cells had the filaments with the number ranged from 1 to 3 with an average of 1.2 ± 0.9 while the remaining 20% had no filaments (Fig. 5b). The average filament length was about half of the wild-type (Fig. 5c). About 80% of the ∆fliH-fliI flhB* *flhA*(F459A) mutant cells produced the filaments with the number ranged from 1 to 4 with an average of 1.3 ± 1.0 (Fig. 5b). The average filament length was one third of the wild-type and was shorter than that of the ∆fliH-fliI flhB* mutant (Fig. 5c). These results indicate that the FlhA(F459A) mutation does not improve the probability of filament assembly at the tip of the hook structure. We obtained essentially the same results with the ∆fliH-fliI flhB* *flhA*(D456V) and ∆fliH-fliI flhB* *flhA*(T490M) mutants (Fig. S3). More than 95% of the *flhB*(P28T) (flhB*) and *flhA*(F459A) mutant cells produced the filaments at the wild-type level (Fig. 5b). The average filament length of the flhB* mutant was almost the same as that of wild-type cells. The average filament length of the *flhA*(F459A) mutant was about half of the wild-type (Fig. 5c) due to the reduced secretion level of FliC by about 4-fold (Fig. 3b, left panel, 1st row, lane 6). Since the FlhA(F459A) mutation increases the substrate specificity switching probability of the flagellar type III protein export apparatus from the hook-type to the filament-type in the ∆fliH-fliI flhB* mutant, thereby increasing the secretion levels of FlgK, FlgL and FliC (Fig. 3b, right panel), these results suggest that newly exported FliC monomers cannot efficiently assemble into the filament at the hook tip in the absence of FliH and FliI.

**Figure 6:**
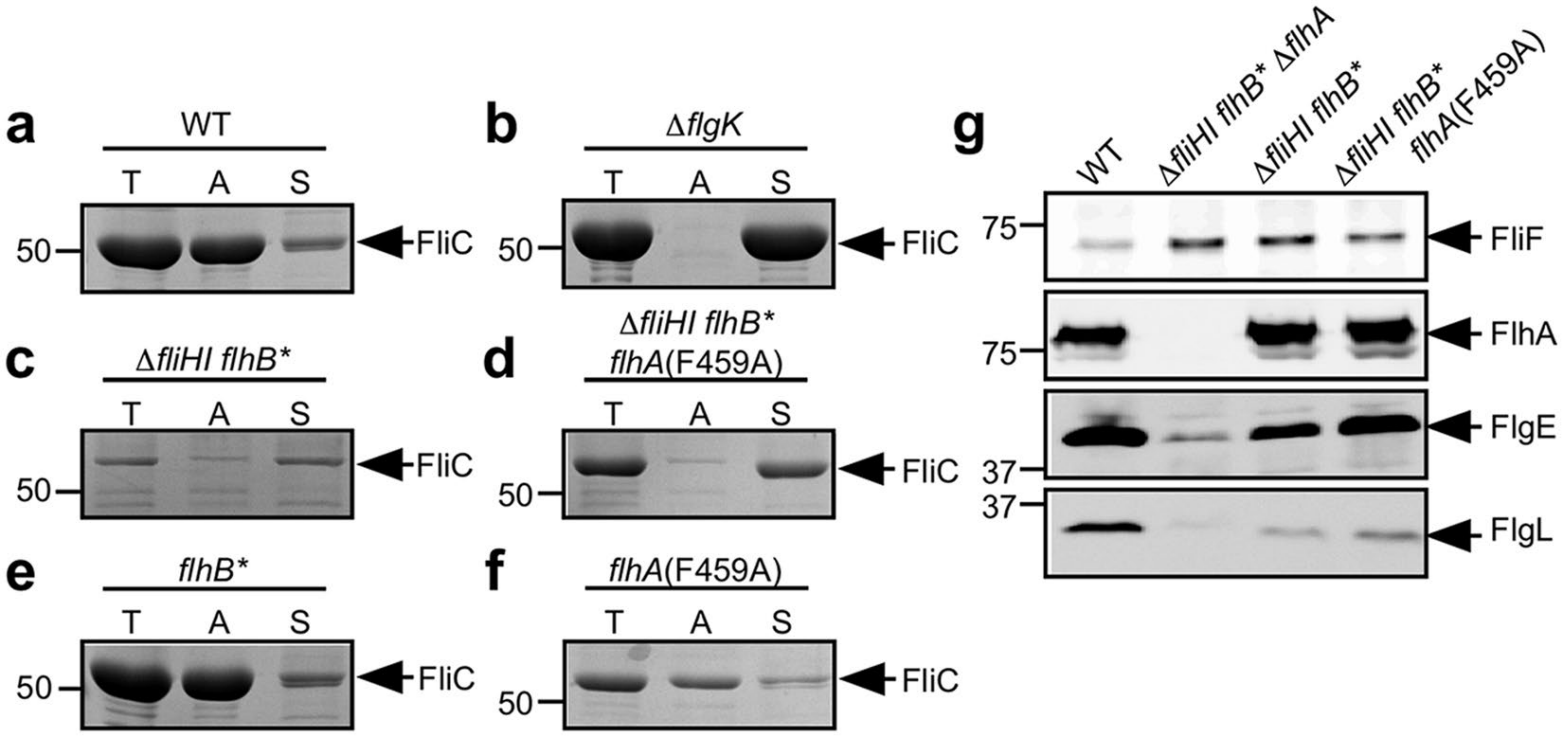
Effect of FliH and FliI deletion on FliC leakage during filament assembly. Measurements of FliC monomers leaked out into the culture media. CBB-staining SDS gels of total extracellular FliC (indicated as T), polymerized FliC (indicated as A), and FliC leaked into the culture media (indicated as S) of (**a**) SJW1103 (WT), (**b**) MM1103gK (∆*flgK*), (**c**) MMHI0117 (∆fliHI flhB*), (**d**) MMHI0117-1 [∆fliHI flhB* flhA(F459A)], (**e**) MMB017 (flhB*) and (**f**) MMA459 [indicated as *flhA*(F459A)]. CBB-stained gels were cropped from original image data shown in Fig. S6 in the Supplemental information. The position of 50 kDa molecular mass marker is indicated on the left. (**g**) Effect of FliH and FliI deletion on assembly of the hook-filament junction at the hook tip. Membrane localization of the MS ring protein, FliF, the transmembrane export gate protein, FlhA, FlgE, and one of the hook-filament junction proteins, FlgL. The membrane fractions of SJW1103, NH004 (∆fliHI flhB* ∆*flhA*), MMHI0117, or MMHI0117-1 were prepared after sonication and ultracentrifugation. Then, the membrane fractions were subjected to SDS-PAGE and were analyzed by immunoblotting with polyclonal anti-FliF, anti-FlhA, anti-FlgE and anti-FlgL antibodies. Positions of FliF, FlhA, FlgE and FlgL are indicated by arrows. Each immunoblot was cropped from its original image data shown in Fig. S7 in the Supplemental information.

To investigate how efficiently exported FliC monomers polymerize into the filament in the ∆fliH-fliI flhB* and ∆fliH-fliI flhB* *flhA*(F459A) mutants, we isolated assembled FliC subunits in the filament form and FliC monomers leaked out into the culture media separately and analyzed them by SDS-PAGE with Coomassie brilliant blue (CBB) staining. In wild-type cells, more than 90% of FliC assembled into the filament, and the remaining 10% existed as monomers in the culture supernatant (Fig. 6a). In contrast, 100% of FliC molecules were monomeric in a *flgK*::Tn10 mutant (Fig. 6b), in agreement with a previous report^41^. In contrast to wild-type cells, more than 90% of FliC subunits existed as monomer in the culture supernatant of the ∆fliH-fliI flhB* and ∆fliH-fliI flhB* *flhA*(F459A) mutants (Fig. 6c and d). We obtained essentially the same results with the ∆fliH-fliI flhB* *flhA*(D456V) and ∆fliH-fliI flhB* *flhA*(T490M) mutants (Fig. S4). Flagellar filament assembly begins with the assembly of the hook-filament junction at the hook tip, followed by the filament cap and finally the filament with the help of the filament cap^42^. Since mutations in FlgK, FlgL and FliD result in leakage of unassembled FliC monomers into the culture media^43^, we suggest that it is the lack of the hook-filament junction and filament cap structures at the hook tip that causes the large amount of FliC monomers leaking into the culture media. This is supported by our observation that much smaller amounts of FlgL were detected in the HBB-containing membrane fractions isolated from the ∆fliH-fliI flhB* and ∆fliH-fliI flhB* *flhA*(F459A) mutants than that from the wild-type, although the levels of the basal-body MS ring protein FliF and FlhA were seen almost at the wild-type levels (Fig. 6g). Since the flhB* and *flhA*(F459A) mutations did not affect FliC leakage by themselves (Fig. 6e and f), we suggest that FlgK, FlgL and FliD require FliH and FliI to efficiently self-assemble at the hook tip prior to filament formation with the help of the FliD cap.

## Discussion

The size and length control of biological nanostructures is one of the most important regulatory processes in living organisms. One example is the hook of the bacterial flagellum. The flagellar type III protein export apparatus switches its substrate specificity upon completion of hook assembly, thereby terminating hook assembly and initiating filament assembly (Fig. 1). At least two flagellar proteins, FliK and FlhB, are responsible for the substrate specificity switch^2,3^. FliK and FlhB_C_ act as a ruler and an export switch, respectively^2,3^. An interaction between FliK_C_ and FlhB_C_ promotes the substrate specificity switch^18,19^, thereby determining the hook length at about 55 nm within 10% error in *Salmonella*^8^. In this study, we showed that the hook length distribution of the ∆fliH-fliI flhB* mutant was much broader than that of wild-type cells and that the FlhA(F459A) mutation increases the secretion levels of FlgE and FliK by about 10-fold and 3-fold, respectively, thereby assisting secreted FliK molecules to more precisely measure the hook length in the ∆fliH-fliI flhB* mutant background (Figs 2 and 3). These suggest that a deletion of FliH and FliI considerably reduces the targeting efficiency of FlgE and FliK to the docking platform of the export gate complex made of FlhA_C_ and FlhB_C_, and that the FlhA(F459A) mutation recovers their targeting efficiency, thereby restoring the hook length control. FliK can measure the hook length only during its infrequent export process because the 1.3 nm channel diameter of the rod and hook is too narrow to permit FlgE export with FliK staying inside the channel^44^. Since the FlhB(P28T) and FlhA(F459A) mutations did not affect the hook length control by themselves (Fig. 2d and e), we propose that FliH and FliI may contribute to hierarchical protein targeting of hook-type export substrates to the FlhA_C_–FlhB_C_ docking platform, allowing the FliK_C_–FlhB_C_ interaction to catalyse the substrate specificity switch of the protein export apparatus at a more appropriate timing of hook assembly.

Over-expression of FlgE causes the ∆fliH-fliI flhB* mutant to produce much longer polyhooks with a length of 190.7 ± 292.0 nm compared to that of wild-type cells over-expressing FlgE (62.0 ± 30.1 nm) (Fig. 4a and c). In contrast, over-expression of FliK reduced the probability of hook formation significantly, thereby reducing the substrate specificity switching efficiency (Fig. S2b) although the length of the hook with the filament attached was 49.5 ± 5.1 nm, which was almost the same as that of wild-type cells over-expressing FliK (48.0 ± 5.1 nm) (Fig. 4b and d). Since we showed that the FlhB(P28T) mutation did not affect the hook length control by itself (Fig. 2d), we propose that FliH and FliI may make the hook length control robust against genetic perturbations.

FlgK and FlgL form the hook-filament junction (11 subunits of FlgK and FlgL per flagellum) at the tip of the hook structure in this order to connect the hook and filament. FliD forms the filament cap (5 subunits of FliD per flagellum) at the tip of the FlgL junction zone to start promoting the assembly of as many as 30,000 FliC molecules into the filament. FlgN, FliS and FliT are flagellar type III export chaperones specific for FlgK and FlgL, FliC and FliD, respectively^3^. These chaperones bind to FlhA_C_ to promote efficient unfolding and transport of their cognate substrates by the export gate complex^14–16^. FliJ binds to FlgN and FliT but not to FliS^45^ and significantly enhances the binding of the FliT/FliD complex to FlhA_C_^14^. Since FlgK, FlgL and FliD are essential for the initiation of filament assembly^42,43^, FlhA_C_ and FliJ are proposed to confer an advantage for efficient export of FlgK, FlgL, and FliD prior to the export of largely excessive amounts of FliC molecules for their assembly at the tip of the junction^14^. In this study, we showed that the ∆fliH-fliI flhB* *flhA*(F459A) mutant cannot form the filaments efficiently (Fig. 5), thereby leaking a large amount of FliC monomers out into the culture media (Fig. 6d). We also showed that the FlhB(P28T) and FlhA(F459A) mutations did not enhance FliC leakage by themselves (Fig. 6e and f). Since much smaller amounts of FlgL were associated with the HBBs of the ∆*fliH-fliI flhB*(P28T) *flhA*(F459A) mutant compared to the wild-type level (Fig. 6g), we suggest that FliH and FliI are required for efficient assembly of FlgK, FlgL and FliD at the hook tip. It has been shown that the binding affinities of FlhA_C_ for the FlgN/FlgK and FliT/FliD complexes are one order of magnitude higher than that for the FliS/FliC complex^16^. Since FlgN and FliT bind to the FliH_2_FliI complex^33,34^ whereas FliS does not^46^, we propose that the FliH_2_FliI complex may contribute to efficient interactions of FlhA with FlgN and FliT more preferentially than with FliS, thereby assuring efficient formation of the junction and cap structures at the hook tip prior to filament formation.

## Methods

### *Salmonella* strains, plasmids, DNA manipulations and media

*Salmonella* strains and plasmids used in this study are listed in Table S1. DNA manipulations and DNA sequencing were carried out as described before^47^. To introduce the FlhA(D456V), FlhA(F459A) or FlhA(T490M) mutation into *Salmonella* ∆fliH-fliI flhB* mutant, the *flhA* gene on the chromosome was replaced by the *flhA*(D456V), *flhA*(F459A) or *flhA*(T490M) allele, using the λ Red homologous recombination system^37^. L-broth^4^, T-broth^35^ and soft agar plates^5^ were prepared as described.

### Preparation of HBBs

*Salmonella* cells were grown in L-broth at 30 °C with shaking until the cell density had reached an OD_600_ of ca. 1.0–1.3. The cells were harvested and suspended in ice-cold 0.1 M Tris-HCl, pH 8.0, 0.5 M sucrose. EDTA and lysozyme were added at the final concentrations of 10 mM and 1.0 mg/ml, respectively. The cell suspensions were stirred for 30 min at 4 °C, and then the cell membranes were solubilized on ice for 1 hour by adding Triton X-100 and MgSO_4_ at final concentrations of 1.0% and 10 mM, respectively. The pH of the cell lysates was adjusted to 10.5 with 5 N NaOH. After centrifugation (10,000 g, 20 min, 4 °C), the lysates were ultracentrifuged (45,000 g, 60 min, 4 °C), and the pellets were resuspended in 10 mM Tris-HCl, pH 8.0, 5 mM EDTA, 1% Triton X-100 and the solution was loaded a 20–50% (w/w) sucrose density gradient in 10 mM Tris-HCl, pH 8.0, 5 mM EDTA, 1% Triton X-100. After sucrose density gradient ultracentrifugation (49,100 g, 13 h, 4 °C), fractions containing intact flagella were collected. After ultracentrifugation at 60,000 g for 60 min, pellets were suspended in 50 mM glycine, pH 2.5, 0.1% Triton X-100, and were incubated at room temperature for 30 min to depolymerize flagellar filaments. After ultracentrifugation, pellets were resuspended in 50 μl of 10 mM Tris-HCl, pH 8.0, 5 mM EDTA, 0.1% Triton X-100. Samples were negatively stained with 2%(w/v) uranyl acetate. Electron micrographs were recorded with a JEM-1011 transmission electron microscope (JEOL, Tokyo, Japan) operated at 100 kV and equipped with a F415 CCD camera (TVIPS, Gauting, Germany) at a magnification of x5,500, which corresponds to 2.75 nm per pixel. Hook length was measured by ImageJ version 1.48 (National Institutes of Health).

### Flagellar protein export assay

Details of sample preparations have been described^48^. Both whole cellular proteins and culture supernatants were normalized to a cell density of each culture to give a constant number of *Salmonella* cells. After SDS-polyacrylamide gel electrophoresis (PAGE), immunoblotting with polyclonal anti-FlgD, anti-FlgE, anti-FliK, anti-FlgK, anti-FlgL or anti-FliC antibody was carried out as described previously^4^. Detection was performed with an ECL prime immunoblotting detection kit (GE Healthcare). Chemiluminescence signals were detected by a Luminoimage analyzer LAS-3000 (GE Healthcare). All image data were processed with Photoshop software CS6 (Adobe). More than five independent experiments were carried out.

### Motility assays in soft agar

Fresh colonies were inoculated onto soft agar plates and incubated at 30 °C. At least seven independent measurements were carried out.

### Measurements the number and length of flagellar filaments

*Salmonella* cells were grown overnight in T-broth containing 100 mM NaCl at 30 °C with shaking. The cells were washed with a motility buffer (10 mM potassium phosphate, pH 7.0, 0.1 mM EDTA, 10 mM L-sodium lactate) and resuspended in the motility buffer. The cells were attached to a cover slip (Matsunami glass, Japan). Flagellar filaments were labeled with Alexa Fluor 594 (Invitrogen) and were observed by fluorescence microscopy as described previously^49^. Fluorescence images were analyzed using ImageJ software version 1.51 (National Institutes of Health) as described previously^50^.

### FliC leakage measurements

*Salmonella* cells were grown with gentle shaking in 5 ml of L-broth at 30 °C until the cell density had reached an OD_600_ of approximately 1.0–1.4. To prepare total extracellular FliC (filaments attached to *Salmonella* cell bodies, filaments detached from the cell body and FliC monomers secreted into culture supernatant), a 1.5 ml of culture was heated at 65 °C for 5 min to depolymerize the filaments into FliC monomers and were centrifuged to obtain cell pellets and culture supernatants, separately. The proteins in the culture supernatants were precipitated by 10% trichloroacetic acid (TCA), suspended in a Tris-SDS loading buffer, and heated at 95 °C for 3 min. To prepare polymerized FliC subunits (filament attached to the cell bodies), a 1.5 ml of culture was centrifuged, and the cell pellets and the culture supernatants were collected, separately. The cell pellets were suspended in 1.5 ml of PBS (8 g of NaCl, 0.2 g of KCl, 3.63 g of Na_2_HPO_4_ 12H_2_O, 0.24 g of KH_2_PO_4_, pH 7.4 per liter) and were heated at 65 °C for 5 min, followed by centrifugation to obtain the cell pellets and supernatants, which contained the cytoplasmic FliC molecules and depolymerized FliC monomers, respectively. Depolymerized FliC monomers were precipitated by 10% TCA, suspended in a Tris-SDS loading buffer, and heated at 95 °C for 3 min. To prepare FliC monomers leaked out into the culture media during filament assembly, the culture supernatants were ultracentrifuged at 85,000 × *g* for 1 hour at 4 °C, and the pellets and the supernatants, which contain flagellar filaments detached from the cell bodies during shaking culture and FliC monomers leaked out into the culture media, respectively, were collected separately. FliC monomers in the supernatants were precipitated by 10% TCA, suspended in the Tris/SDS loading buffer and heated at 95 °C for 3 min. Samples were analyzed by SDS-PAGE with Coomassie Brilliant Blue (CBB) staining. Gel images were captured by a Luminoimage analyzer LAS-3000 (GE Healthcare). All image data were processed with Photoshop software CS6 (Adobe). At least three independent experiments were performed.

### Fractionation of cell membrane

Cells were grown exponentially in 30 ml L-broth at 30 °C with shaking. The cells were harvested, resuspended in 3 ml PBS, and sonicated. After the cell debris was removed by low-speed centrifugation, the cell lysates were centrifuged (100,000 g, 30 min, 4 °C). After carefully removing the soluble fractions, membranes were resuspended in 300 μl of SDS-loading buffer. The protein concentration was normalized to an optical density of OD_600_ and heated at 95 °C for 5 min. After the membrane proteins in each fraction were separated by SDS-PAGE, immunoblotting with polyclonal anti-FlgE, anti-FlgL, anti-FlhA or anti-FliF antibody was carried out. At least three independent experiments were carried out.

### Data availability

All data generated or analysed during this study are included in this published article and its Supplementary Information files.

## Electronic supplementary material

Supplementary Information
