# Supplementary material for: Novel insights into the mechanism of well-ordered assembly of bacterial flagellar proteins in Salmonella
Source: Sci Rep. 2018 Jan 29;8:1787. doi: 10.1038/s41598-018-20209-3 (PMC5789064; doi:10.1038/s41598-018-20209-3)
Supplement: Supplementary file 1 — Supplementary Information [file 41598_2018_20209_MOESM1_ESM.pdf]

Supporting Information

Novel insights into the mechanism of well-ordered assembly of bacterial flagellar proteins in *Salmonella*

Yumi Inoue, Yusuke V. Morimoto,

Keiichi Namba and Tohru Minamino

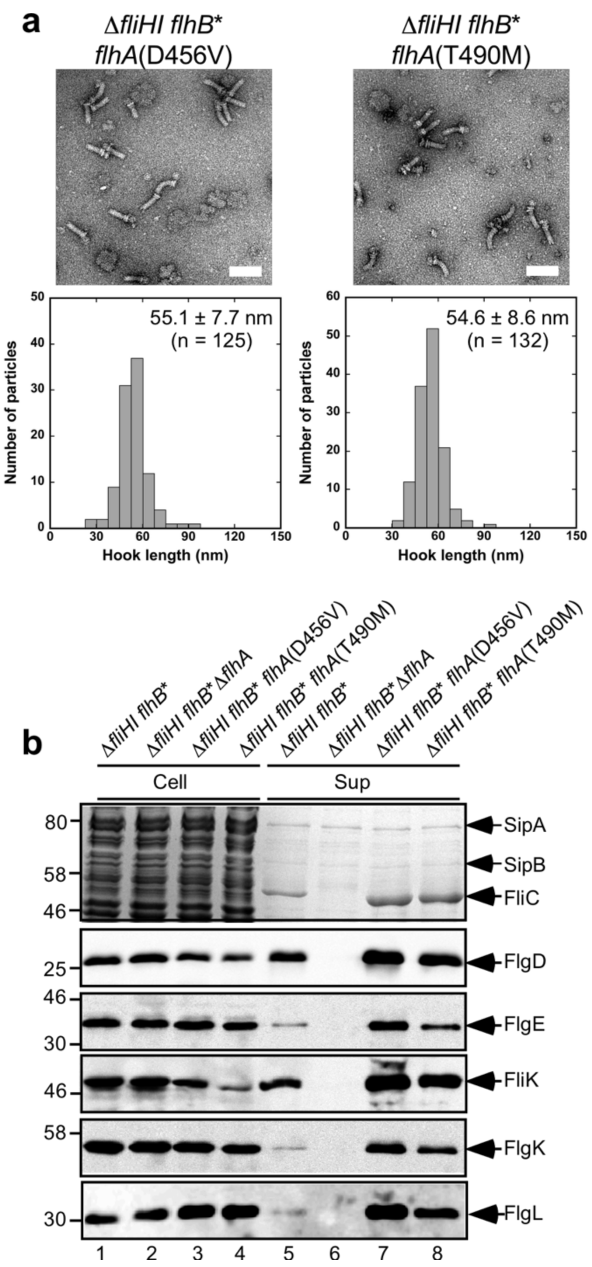

Fig. S1. Effect of the FlhA(D456A) and FlhA(T490M) mutations on flagellar protein export and assembly. (a) Electron micrographs of HBBs and histograms of hook length distribution of MMHI0117-2 [Δ*fliHI flhB* flhA*(D456V)] and MMHI0117-3 [Δ*fliHI flhB* flhA*(T490M)] (b) Flagellar protein export assays. Whole cell proteins (Cell) and culture supernatant fractions (Sup) were prepared from MMHI0117 (indicated as Δ*fliHI flhB**), NH004 (indicated as Δ*fliHI flhB** Δ*flhA*), MMHI0117-2 and MMHI0117-3 and were normalized to their optical density of OD_{600} to give a constant number of cells. 8 μl of each sample was subjected to SDS-PAGE and analyzed by CBB staining (1st row) and immunoblotting, using polyclonal anti-FlgD (2nd row), anti-FlgE (3rd row), anti-FliK (4th row), anti-FlgK (5th row) or anti-FlgL (6th row) antibody. The positions of molecular mass markers (KDa) are indicated on the left. SipA and SipB, which are secreted by the SPI-1 virulence-associated type III secretion apparatus, serve as loading controls.

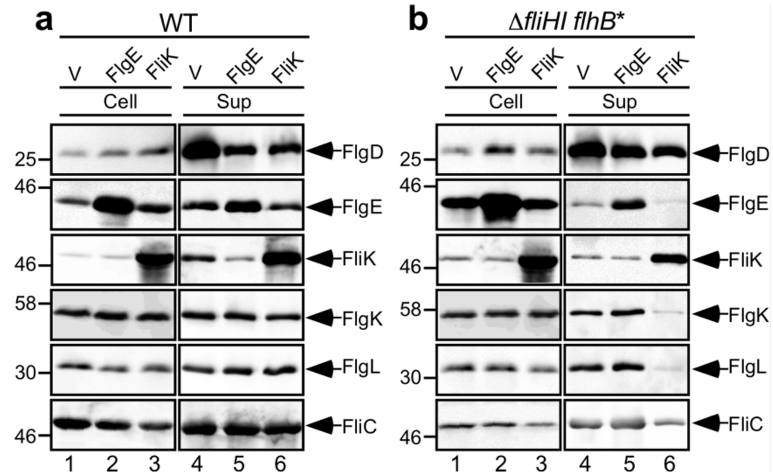

Fig. S2. Effect of over-expression of FlgE and FliK on flagellar protein export in the presence and absence of FliH and FliI. Whole cell proteins (Cell) and culture supernatant fractions (Sup) were prepared from (a) SJW1103 (indicated as WT) carrying pTrc99A (V), pNM001 (FlgE) or pMMIK100 (FliK) and (b) MMHI0117 (indicated as Δ*fliHI flhB**) harbouring pTrc99A (V), pNM001 (FlgE) or pMMIK100 (FliK) and were normalized to their optical density of OD_{600} to give a constant number of cells. 8 μl of each sample was subjected to SDS-PAGE and analyzed by immunoblotting, using polyclonal anti-FlgD (1st row), anti-FlgE (2nd row), anti-FliK (3rd row), anti-FlgK (4th row), anti-FlgL (5th row) or anti-FliC (6th row) antibody. The positions of molecular mass markers (KDa) are indicated on the left.

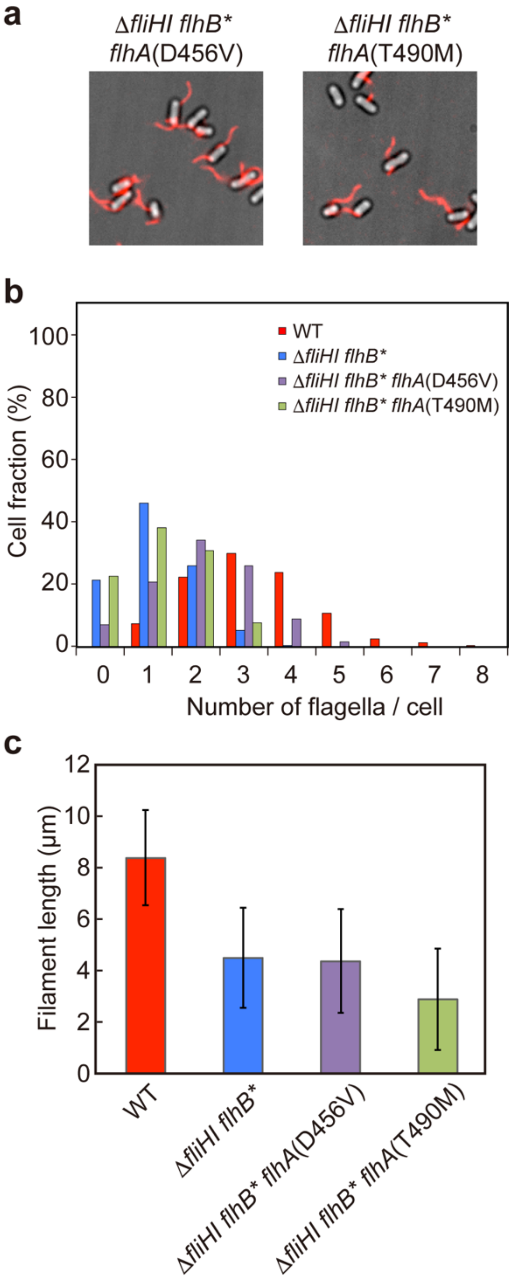

Fig. S3. Effect of the FlhA(D456V) and FlhA(T490M) mutations on filament assembly in the Δ*fliH-fliI flhB(P28T)* mutant background. (a) Fluorescent images of MMHI0117-2 [Δ*fliHI flhB* flhA*(D456V)] and MMHI0117-3 [Δ*fliHI flhB* flhA*(T490M)]. Flagellar filaments were labeled with Alexa Fluor 594. The fluorescence images of the filaments labeled with Alexa Fluor 594 (red) were merged with the bright field images of the cell bodies. (b) Distribution of the number of the flagellar filaments. More than 200 cells for each strain were counted. (c) Measurements of the length of the flagellar filaments. Filament length is the average of more than 100 cells for each strain, and vertical lines are standard deviations.

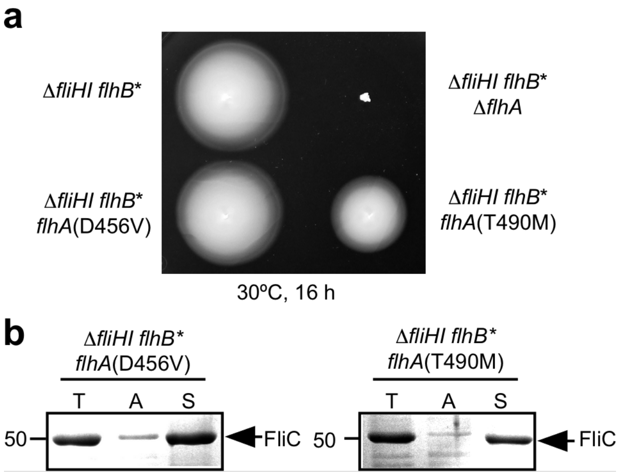

Fig. S4. Effect of the FlhA(D456V) and FlhA(T490M) mutations on motility of the *ΔfliH-fliI flhB(P28T)* mutant. (a) Motility of MMHI0117 (indicated as Δ*fliHI flhB**), NH004 (indicated as Δ*fliHI flhB** Δ*flhA*), MMHI0117-2 [Δ*fliHI flhB* flhA*(D456V)] and MMHI0117-3 [Δ*fliHI flhB* flhA*(T490M)] in soft agar. Plates were incubated at 30ºC for 16 hours. (b) Measurements of FliC leakage during flagellar filament assembly. CBB-staining gels of total extracellular FliC (indicated as T), polymerized FliC (indicated as A), and secreted FliC (indicated as S) of MMHI0117-2 and MMHI0117-3. A position of the 50 kDa molecular mass marker is indicated on the left. The position of 50 KDa molecular mass marker is indicated on the left.

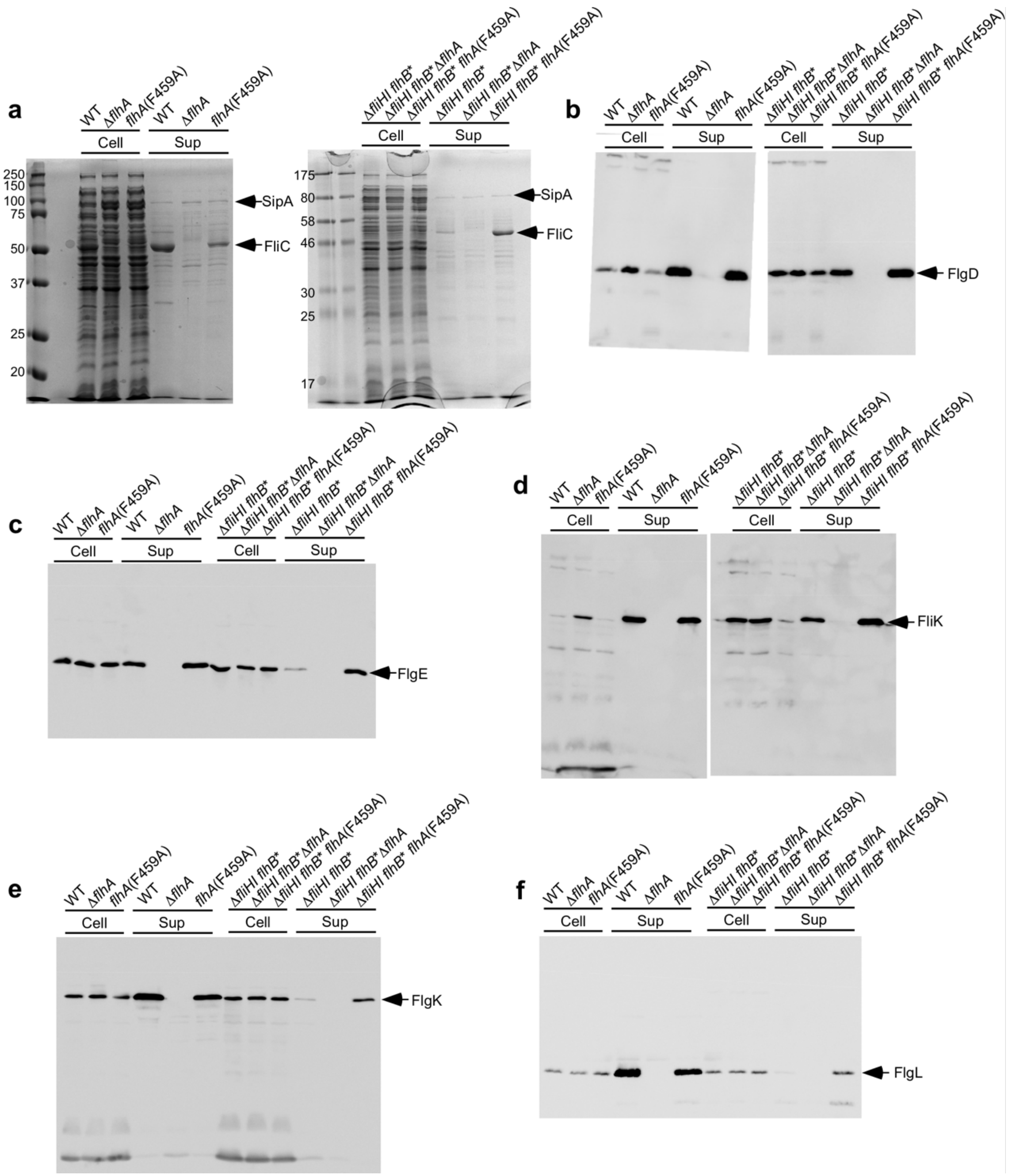

Fig. S5. Original image data of Figure 3b. Whole cell proteins (Cell) and culture supernatant fractions (Sup) were prepared from SJW1103 (indicated as WT), NH001 (indicated as Δ*flhA*), MMA459 [indicated as *flhA*(F459A)], MMHI0117 (indicated as Δ*fliHI flhB**), NH004 (indicated as Δ*fliHI flhB** Δ*flhA*) and MMHI0117-1 [Δ*fliHI flhB** *flhA*(F459A)]. 8 μl of each protein sample, which was normalized to an optical density of OD_{600}, was subjected to SDS-PAGE and was analyzed by (a) CBB staining and immunoblotting with polyclonal (b) anti-FlgD, (c) anti-FlgE, (d) anti-FliK, (e) anti-FlgK or (f) anti-FlgL antibody. All image data were captured by a Luminoimage analyser LAS3000. The positions of molecular mass markers (kDa) are indicated on the left.

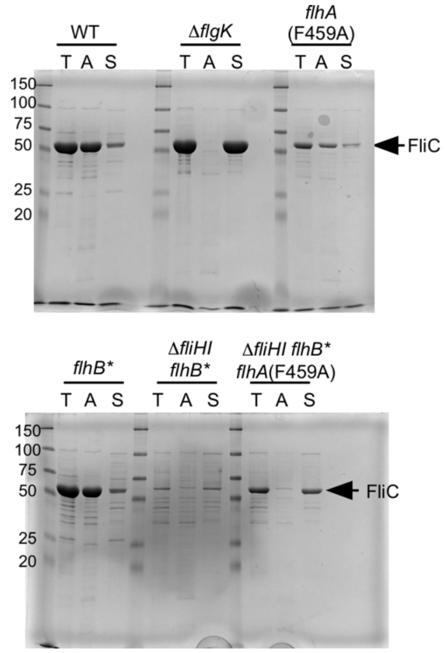

Fig. S6. Original image data of panels a–f in Figure 6. CBB-staining SDS gels of total extracellular FliC (indicated as T), polymerized FliC (indicated as A), and FliC leaked into the culture media (indicated as S) of SJW1103 (indicated as WT), MM1103gK (indicated as Δ*flgK*), MMA459 [indicated as *flhA*(F459A)], MMB017 (*flhB**), MMHI0117 (indicated as Δ*fliHI flhB**) and MMHI0117-1 [Δ*fliHI flhB* flhA*(F459A)]. Gel images were captured by a Luminoimage analyser LAS3000.

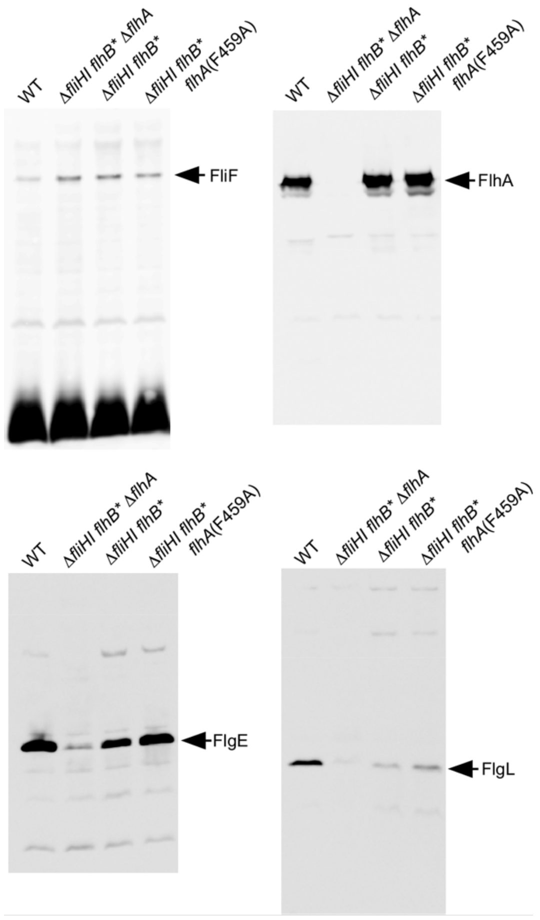

Fig. S7. Original image data of Figure 6g. The membrane fractions of SJW1103, NH004 (indicated as Δ*fliHI flhB** Δ*flhA*), MMHI0117, or MMHI0117-1 were prepared after sonication and ultracentrifugation. Then, the membrane fractions were subjected to SDS-PAGE, and analyzed by immunoblotting with polyclonal anti-FliF, anti-FlhA, or anti-FlgL antibody. Chemiluminescence signals were detected by a Luminoimage analyzer LAS-3000.

Table S1. Plasmids and *Salmonella* strains used in this study

Plasmids/ Strains	Relevant characteristics	Source or reference
**Plasmids**		
pTrc99AFF4	Modified pTrc vector	1
pMMIK100	pTrc99AFF4/ FliK	2
pNM001	pTrc99AFF4/ FlgE	3
Salmonella		
SJW1103	Wild type for motility and chemotaxis	4
MM1103gK	*flgK*::Tn*10*	5
MMHI0117	Δ*fliH-fliI flhB*(P28T)	6
MMB017	*flhB*(P28T)	6
MMA459	*flhA*(F459A)	This study
MMHI0117-1	Δ*fliH-fliI flhB*(P28T) *flhA*(F459A)	This study
MMHI0117-2	Δ*fliH-fliI flhB*(P28T) *flhA*(D456V)	This study
MMHI0117-3	Δ*fliH-fliI flhB*(P28T) *flhA*(T490M)	This study
NH001	Δ*flhA*	7
NH004	Δ*fliH-fliI flhB*(P28T) Δ*flhA*	7

References

1. Ohnishi, K., Fan, F., Schoenhals, G.J., Kihara, M. & Macnab R.M. The FliO, FliP, FliQ, and FliR proteins of *Salmonella typhimurium*: putative components for flagellar assembly. *J. Bacteriol.* **179**, 6092–6099 (1997).
2. Minamino, T., Ferris, H.U., Morioya, N., Kihara, M. & Namba, K. Two parts of the T3S4 domain of the hook-length control protein FliK are essential for the substrate specificity switching of the flagellar type III export apparatus. *J. Mol. Biol.* **362**, 1148–1158 (2006).
3. Moriya, N., Minamino, T., Hughes, K. T., Macnab, R. M. & Namba, K. The type III flagellar export specificity switch is dependent on FliK ruler and a molecular clock. *J. Mol. Biol.* **359**, 466–477 (2006).
4. Yamaguchi, S., Fujita, H., Sugata, K., Taira, T. & Iino, T. Genetic analysis of *H2*, the structural gene for phase-2 flagellin in *Salmonella*. *J. Gen. Microbiol.* **130**, 255–265 (1984).
5. Renault, T. T. *et al*. Bacterial flagella grow through an injection-diffusion mechanism. *eLife* **6**, e23136 (2017).
6. Minamino, T. & Namba, K. Distinct roles of the FliI ATPase and proton motive force in bacterial flagellar protein export. *Nature* **451**, 485–488 (2008).
7. Hara, N., Namba, K. & Minamino, T. Genetic characterization of conserved charged residues in the bacterial flagellar type III export protein FlhA. *PLOS One* **6**, e22417 (2011).
